# Accuracy of PE rule-out strategies in pregnancy: secondary analysis of the DiPEP study prospective cohort

**DOI:** 10.1136/emermed-2019-209213

**Published:** 2020-04-09

**Authors:** Steve Goodacre, Catherine Nelson-Piercy, Beverley J Hunt, Gordon Fuller

**Affiliations:** 1 School of Health and Related Research, University of Sheffield, Sheffield, UK; 2 Women’s Health Academic Centre, Guy’s & St Thomas’s NHS Foundation Trust, London, UK; 3 Departments of Haematology and Rheumatology, Guy’s & St Thomas’s NHS Foundation Trust, London, UK

**Keywords:** pulmonary embolism, thrombo-embolic disease, diagnosis, diagnosis

## Abstract

**Objective:**

Recent studies suggest that combinations of clinical probability assessment (the YEARS algorithm or Geneva score) and D-dimer can safely rule out suspected pulmonary embolism (PE) in pregnant women. We performed a secondary analysis of the DiPEP (Diagnosis of Pulmonary Embolism in Pregnancy) study data to determine the diagnostic accuracy of these strategies.

**Methods:**

The DiPEP study prospectively recruited and collected data and blood samples from pregnant/postpartum women with suspected PE across 11 hospitals and retrospectively collected data from pregnant/postpartum women with diagnosed PE across all UK hospitals (15 February 2015 to 31 August 2016). We selected prospectively recruited pregnant women who had definitive diagnostic imaging for this analysis. We used clinical data and D-dimer results to determine whether the rule out strategies would recommend further investigation. Two independent adjudicators used data from imaging reports, treatments and adverse events up to 30 days to determine the reference standard.

**Results:**

PEs were diagnosed in 12/219 (5.5%) women. The YEARS/D-dimer strategy would have ruled out PE in 96/219 (43.8%) but this would have included 5 of the 12 with PEs. Sensitivity for PE was 58.3% (95% CI 28.6% to 83.5%) and specificity 44.0% (37.1% to 51.0%). The Geneva/D-dimer strategy would have ruled out PE in 46/219 (21.0%) but this would have included three of the 12 with PE. Sensitivity was 75.0% (95% CI 42.8% to 93.3%) and specificity 20.8% (95% CI 15.6% to 27.1%). Administration of anticoagulants prior to blood sampling may have reduced D-dimer sensitivity for small PE.

**Conclusion:**

Strategies using clinical probability and D-dimer have limited diagnostic accuracy and do not accurately rule out all PE in pregnancy. It is uncertain whether PE missed by these strategies lead to clinically important consequences.

**Key messages**

**What is already known on this subject**

- PE is an important potential cause of morbidity in pregnancy but symptoms suggesting PE are common in pregnancy.
- Recent studies suggest that a combination of clinical probability assessment and D-dimer measurement can allow safe discharge without imaging in a proportion of pregnant women with suspected PE.

**What this study adds**

- This secondary analysis of the DiPEP study showed that strategies using clinical probability and D-dimer have limited diagnostic accuracy and do not accurately rule out all PE in pregnancy. It is uncertain whether PE missed by these strategies lead to clinically important consequences.

## Introduction

Recent studies suggest that combinations of clinical probability assessment and D-dimer can safely rule out pulmonary embolism (PE) in a proportion of pregnant women presenting with suspected PE who would otherwise require imaging to rule out PE. van der Pol *et al* 1 tested a pregnancy-modified YEARS algorithm and D-dimer in 498 pregnant women with suspected PE presenting to 18 hospitals over 5 years (20 with PE), ruling out PE without scan or adverse outcome in 195 (39%). Righini *et al* 2 tested clinical probability scoring with the Geneva score alongside D-dimer in 395 pregnant women with suspected PE presenting to eleven hospitals over 8 years (28 with PE), ruling out PE without scan or adverse outcome in 46 (12%).

These studies suggest that a proportion of women can avoid imaging for PE but may lack statistical power to ensure an acceptably low rate of adverse outcome in this group. The studies were designed to detect 3-month event rates of 2.7% and 3.0%, respectively, but it is unclear whether patients and clinicians consider these event rates acceptable for a potentially catastrophic outcome. We are not aware of any studies estimating clinician or patient willingness to accept risk of adverse outcome after discharge for suspected PE in pregnancy but a survey of emergency physicians reported that only 18/1023 (1.8%) considered an adverse event rate above 2% acceptable following negative assessment for acute chest pain.^3^ Furthermore, the sample size was estimated and event rates reported across the entire cohort, including those who received imaging, whereas the relevant population is those who did not receive imaging. The upper 95% CI for the zero event rates in the non-imaged population are 2.4% for the 195 women in the van der Pol study and 9.6% in the 46 women in the Righini study.

The DiPEP (Diagnosis of Pulmonary Embolism in Pregnancy) study prospectively recruited and collected data and blood samples from pregnant and postpartum women with suspected PE across 11 sites, and retrospectively collected data from pregnant and postpartum women with diagnosed PE across the whole of the UK.^4 5^ Clinical probability assessment and D-dimer were not routinely used to select women for investigation in the study and the relevant guidelines recommended against this practice.^6^ The DiPEP data, therefore, offered the opportunity to determine the diagnostic accuracy of rule out strategies, compared with a reference standard based on radiological imaging.

We aimed to undertake a secondary analysis of data and blood samples from pregnant women with suspected PE who were prospectively recruited to the DiPEP study, to determine the accuracy of rule out strategies based on clinical probability assessment and D-dimer.

## Methods

DiPEP was a prospective cohort study augmented with additional retrospective cases to determine whether clinical features, individually or in the form of a clinical decision rule, or D-dimer could be used to select pregnant and postpartum women for diagnostic imaging.^4^ It also involved analysis of blood samples collected from the prospective cohort to determine the accuracy of a range of biomarkers for PE in pregnancy and post partum.^5^

Prospectively recruited pregnant women with suspected PE were selected for this analysis. Women were identified at emergency departments and maternity units across eleven sites over 18 months and asked to provide written informed consent to data and blood sample collection. A research nurse or midwife then completed a case report form, including details of patient characteristics, investigations and treatments for venous thromboembolism (VTE). Hospital records were reviewed at 30 days after recruitment and any subsequent VTE-related investigations, treatments or adverse events were recorded. All management was at the discretion of the attending clinician and determined on the basis of patient need.

We excluded retrospectively identified women with diagnosed PE from this analysis because blood samples could not be collected from these women; and to prevent selection bias from incomplete retrospective case ascertainment, and avoid information bias from retrospective data abstraction from case notes. Results of hospital D-dimer testing were available for some but different assays and thresholds for positivity were used in each hospital. Prospectively recruited postpartum women were excluded because neither of the rule out strategies had been developed or evaluated for postpartum women.

The pregnancy-adapted YEARS algorithm used three criteria to select women for D-dimer testing: clinical signs of deep vein thrombosis (DVT), haemoptysis and PE as the most likely diagnosis. The DiPEP case report form recorded whether haemoptysis was a presenting symptom, whether there were clinical signs of DVT and what was considered the most likely diagnosis after clinical assessment. These data were used to determine the results of applying the three criteria to DiPEP patients.

Righini *et al* 2 used the revised Geneva score (low or intermediate pretest clinical probability) to select women for D-dimer testing. The DiPEP case report form recorded age, heart rate, whether haemoptysis was a presenting symptom any previous history of VTE, surgery or significant injury in the previous 4 weeks, pre-existing cancer and clinical signs of DVT. A free-text box for recording any other symptoms was searched to identify patients presenting with unilateral lower limb pain. These data were used to determine the results of applying the revised Geneva score to DiPEP patients. We have previously evaluated a pregnancy-modified version of the revised Geneva score^4^ but for this analysis used an unmodified version to ensure consistency with the study of Righini *et al*.^2^

Serum and citrate blood samples were collected by a member of the clinical team or research nurse/midwife using good venepuncture technique, ideally while obtaining routine blood samples for standard clinical assessment in diagnostic workup. The samples were centrifuged at 2000 g for 15 min at room temperature and frozen down within 4 hours of being obtained. Citrate samples were further processed to obtain platelet free plasma.

Plasma and serum samples were stored in aliquots labelled with the patient ID and the storage box coordinates recorded on paper and electronic study documentation, according to local protocols. The samples were stored in −70°C freezers at each participating hospital (with the exception of one location where a −40°C freezer was used) for the duration on the study until all samples were transported for analysis to Guy’s St Thomas Trust, London, UK.

The Zymutest D-Dimer Eliza assay (Quadratech Diagnostics, Epsom, UK) was used to measure the D-Dimers by ELISA according to the manufacturer’s instructions. The recommended threshold for the test was 400 ng/mL. The coefficient of variation was 4.6% intra-assay and 10.8% inter assay for the Zymutest D-Dimer. In accordance with the strategy of Righini *et al*, we used the recommended threshold of 400 ng/mL to rule out PE in women with a low or intermediate revised Geneva score. In accordance with the diagnostic strategy tested by van der Pol *et al*, we used the recommended threshold for positivity of 400 ng/mL to rule out PE in women when one or more of the YEARS criteria and 800 ng/mL (double the recommended threshold for positivity) when none of the YEARS criteria were met.

The reference standard was determined by two independent observers, who reviewed reports of VTE-related diagnostic imaging, treatments and adverse events up to 30 days after recruitment. Any disagreements were settled by a third independent expert. The reviewers were blinded to index test results.

In accordance with the primary analysis plan for DiPEP, this analysis was limited to those with conclusive imaging. This meant that the reference standard was effectively based on imaging results alone. A secondary analysis explored the effect of including those with inconclusive or no imaging. Analysis was descriptive, involving estimation of the sensitivity and specificity of each strategy for diagnosing PE, with a 95% CI.

### Patient and public involvement

The DiPEP study steering committee included representatives from two patient organisations, Thombosis UK and the Sheffield Emergency Care Forum. They advised on the design, conduct and interpretation of the study, and assisted with dissemination of the findings.

## Results

**Figure 1:**
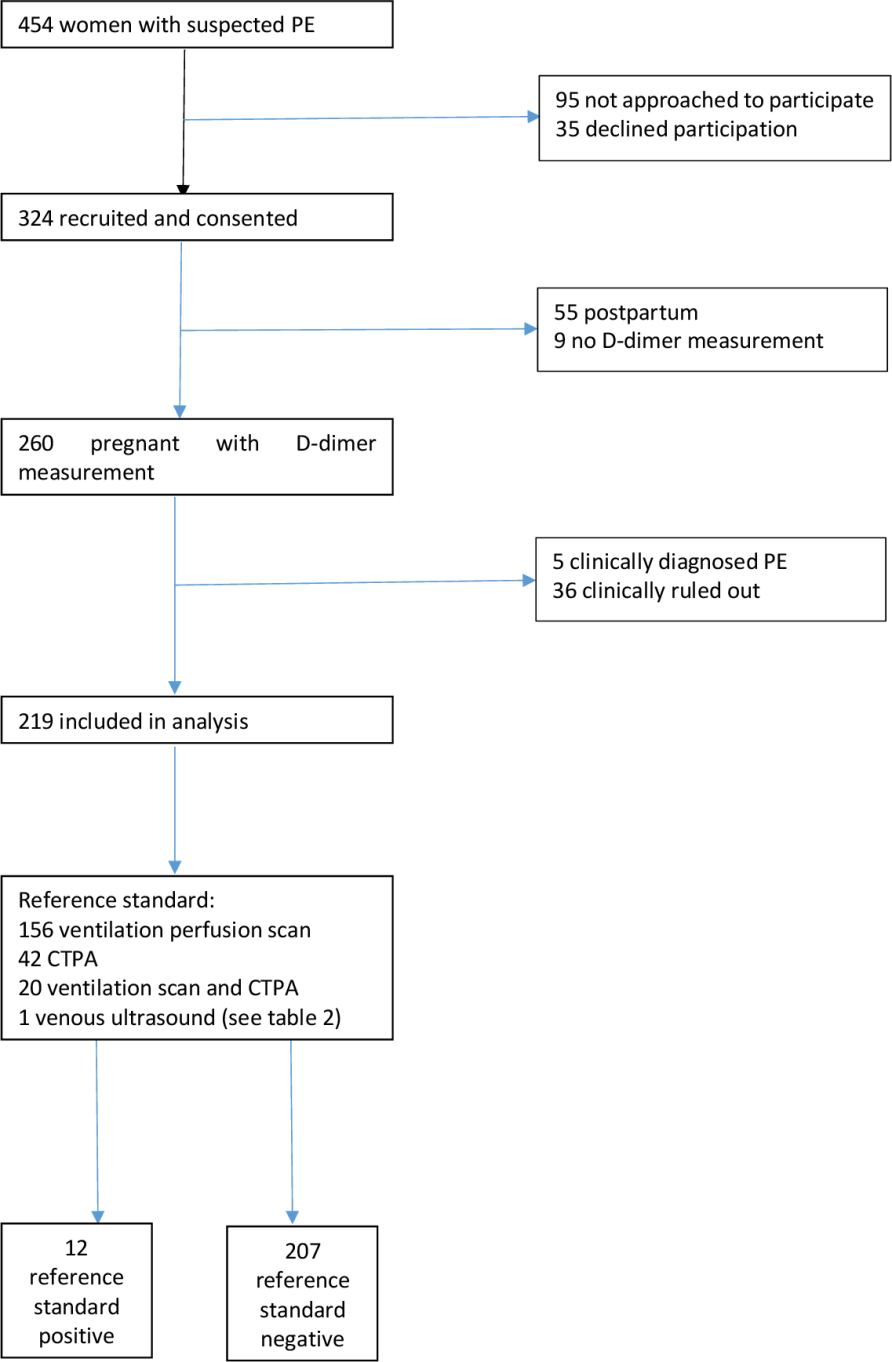
Flow of participants through the study. PE, pulmonary embolism; CTPA, computed tomography pulmonary angiogram

A total of 324 women were recruited across 11 participating sites between 15 February 2015 and 31 August 2016. We excluded 55 who were post partum and a further 9 who did not have D-dimer measurements recorded. The analysis was limited to 219 women with conclusive imaging (five were excluded with clinically diagnosed PE and 36 with clinically ruled out PE). Figure 1 shows the flow of participants through the study and table 1 outlines the characteristics of those included in the analysis.

**Table 1:** Characteristics of the study population

Characteristic	Patients (n=219)
Mean age	29.3 years
Mean BMI	27.9 kg/m^2^
Mean heart rate	97.8/min
Mean respiratory rate	18.9/min
Mean oxygen saturation	97.8%
Mean systolic blood pressure	120.7 mm Hg
Mean diastolic blood pressure	71.9 mm Hg
Mean temperature	36.5°C
Smoking status	
Never	150 (68.5%)
Gave up before	28 (12.8%)
Gave up during	15 (6.8%)
Current	26 (11.9%)
One or more previous pregnancies <24 weeks	86 (39.3%)
One or more previous pregnancy >24 weeks	137 (62.6%)
Family history of thrombosis	41 (18.7%)
History of varicose veins	18 (8.2%)
History of intravenous drug use	1 (0.5%)
Known thrombophilia	6 (2.7%)
Surgery in previous 4 weeks	1 (0.5%)
Significant injury in the previous 4 weeks	1 (0.5%)
History of thrombosis	17 (7.8%)
First trimester	20 (9.1%)
Second trimester	81 (37.0%)
Third trimester	118 (53.9%)
Multiple pregnancy	11 (5.0%)
Long-haul travel during pregnancy	20 (9.1%)
Three or more days of immobility/bed rest	13 (5.9%)

BMI, body mass index.

PE was diagnosed in 12/219 (5.5%). Table 2 shows the maternal and gestational age for each woman with PE and how PE was diagnosed on imaging. The diagnosis was based on computed tomography pulmonary angiogram (CTPA) findings for five women, ventilation-perfusion scanning for six women, and lower limb ultrasound evidence of DVT in the presence of symptoms of PE for one woman.

**Table 2:** Characteristics of the women diagnosed with PE

Patient no	Maternal age	Gestational age	Imaging method	Imaging report
1	35	28/40	CTPA	Segmental PE lower left lobe
2	34	37/40	VQ	Isolated wedge shaped perfusion defect in the apical segment of the right lower lobe
3	26	36/40	CTPA	Extensive bilateral PE
4	33	35/40	CTPA	On balance of probability non-occlusive filling defect in the left upper lobe segmental vessel represents PE
5	26	23/40	VQ	Perfusion defects in both lungs
6	33	24/40	VQ	Extensive reduction of perfusion to the right lung with further segmental areas of perfusion loss in the left lung. The ventilation scan is almost normal.
7	26	32/40	US lower limb	Echogenic thrombus demonstrated within the left common femoral vein, which extends down to the left popliteal vein. The vein was not patent and incompressible
8	21	29/40	CTPA	Appearances are highly suspicious for solitary small PE
9	33	24/40	VQ	Unmatched perfusion defect in the right mid to lower lung
10	32	27/40	VQ	Appearances are in keeping with a right acute PE
11	25	15/40	VQ	The segmental perfusion defect in the right lung posteriorly is mismatched with the ventilation images. Findings are in line with PE
12	34	13/40	CTPA	Extensive bilateral pulmonary embolus

Table 3 shows the results of applying the YEARS/D-dimer (van der Pol) strategy to this cohort and table 4 shows the results of applying Geneva/D-dimer (Righini) strategy. The YEARS/D-dimer strategy would have resulted in 96/219 women (43.8%) being discharged without imaging, but this would have included 5/12 with PE. The sensitivity of the strategy was 58.3% (95% CI 28.6% to 83.5%) and specificity was 44.0% (37.1% to 51.0%). The Geneva/D-dimer strategy would have resulted in 46/219 women (21.0%) being discharged without scanning, but this would have included 3/12 with PE. The sensitivity of the strategy was 75.0% (42.8% to 93.3%) and specificity was 20.8% (15.6% to 27.1%).

**Table 3:** Results of applying the YEARS/D-dimer strategy (N, %, 95% CI)

	PE	No PE	Total
Strategy positive	7 58.3 28.6 to 83.5	116 56.0 49.0 to 62.9	123 56.2 49.3 to 62.8
Strategy negative	5 41.7 16.5 to 71.4	91 44.0 37.1 to 51.0	96 43.8 37.2 to 50.7
Total	12	207	219

PE, pulmonary embolism.

**Table 4:** Results of applying the Geneva/D-dimer strategy (N, %, 95% CI)

	PE	No PE	Total
Strategy positive	9 75.0 42.8 to 93.3	164 79.2 72.9 to 84.4	173 79.0 72.9 to 84.1
Strategy negative	3 25.0 6.7 to 57.2	43 20.8 15.6 to 27.1	46 21.0 15.9 to 27.1
Total	12	207	219

PE, pulmonary embolism.

Secondary analysis including those with inconclusive or no imaging produced similar results. The YEARS/D-dimer strategy sensitivity was 52.9% (28.5% to 76.1%) and specificity 42.4% (36.1% to 48.9%). The Geneva/D-dimer strategy sensitivity was 70.6% (44.1% to 88.6%) and specificity 19.8% (15.1% to 25.4%).

Table 5 shows the elements of each strategy and whether the overall strategy indicated PE (requiring imaging) or no PE (no imaging required). The YEARS algorithm was positive in seven women and negative in five. The Geneva score was high in two women, intermediate in nine women and low in one. D-dimer was positive in eight women using the conventional threshold and positive in six women using a threshold double the conventional threshold.

**Table 5:** Application of the rule out strategies to women with PE

Patient	Haemoptysis	Clinical signs DVT	PE most likely diagnosis	Geneva score	D-dimer	Thrombo-prophylaxis	Anticoagulant (time before D-dimer)	YEARS/D-dimer strategy	Geneva/D-dimer strategy
1	No	Yes	No	12	708	No	Dalteparin 7500 IU, 14 hours	PE	PE
2	No	No	No	5	1469	No	Dalteparin 6000 IU, 2 hour	PE	PE
3	No	No	Yes	5	4802	Yes	Tinzaparin 19 000 IU, 16 hours	PE	PE
4	No	No	No	6	261	Yes	No*	No PE	No PE
5	No	No	No	0	823	No	Tinzaparin 175 u/kg, 0 hour	PE	PE
6	No	No	No	5	1444	No	Dalteparin 7500 IU, 4.5 hours	PE	PE
7	No	Yes	Yes	9	2696	No	Dalteparin 7500 IU, 7 hours	PE	PE
8	No	No	No	5	662	No	Dalteparin 10 000 IU, 7 hours	No PE	PE
9	No	Yes	No	15	194	Yes	Dalteparin 12 500 IU, 2 hours	No PE	PE
10	Yes	No	No	5	265	Yes	Enoxaparin 80 mg, 6.5 hours	No PE	No PE
11	No	No	Yes	5	263	No	No	No PE	No PE
12	No	No	Yes	5	4329	No	Tinzaparin 18 000 IU, 13.5 hour	PE	PE

*Recorded as receiving no anticoagulation before blood sampling, but also recorded as commencing tinazaparin 15 000 IU daily for thromboprophylaxis 3 months prior to presentation.

DVT, deep vein thrombosis; PE, pulmonary embolism.

Table 5 also shows whether the women with PE received anticoagulation prior to blood sampling, as this may interfere with D-dimer measurement. Ten women received anticoagulation, ranging from 0 to 16 hours before sampling. One woman was recorded as having no anticoagulation prior to blood sampling but elsewhere was recorded as commencing thromboprophylaxis 3 months prior to presentation. It is therefore not clear whether her D-dimer result could have been influenced by anticoagulant treatment. One woman had no anticoagulation prior to blood sampling and, although PE was considered the most likely diagnosis and the Geneva score indicated intermediate risk of PE, both strategies indicated no PE on account of her negative D-dimer result (263 ng/mL).

## Discussion

### Main findings

Our findings suggest that PE rule out strategies based on clinical probability assessment and D-dimer do not reliably rule out all PE in pregnant women with suspected PE who would otherwise require imaging. The YEARS/D-dimer strategy would have missed five of the 12 and the Geneva/D-dimer strategy would have missed three of the 12 women diagnosed with PE in the DiPEP study.

These findings appear to be inconsistent with the original studies of these algorithms, which identified no symptomatic VTE on follow-up of 46 women with negative assessment using the Geneva/D-dimer strategy and only one DVT on follow-up of 195 women with negative assessment using the YEARS/D-dimer strategy. This apparent inconsistency may be explained by the different designs of the studies, both of which provide useful information and neither of which should be considered definitive. DiPEP is a diagnostic accuracy study in which strategies are compared with an imaging reference standard. The Righini and van der Pol studies are management studies that estimate the risk of adverse outcome in women who have PE ruled out without imaging. Interpretation requires consideration of the strengths and limitations of both designs, and the consequences of missed PE.

The relatively low prevalence of PE in the Righini and van der Pol study cohorts means that even if the strategies missed a quarter of the cases of PE, this would only result in a few missed cases among those discharged without imaging or treatment. The outcomes of PE without treatment are difficult to estimate but it is conceivable that a small number of missed cases could occur without leading to serious adverse outcome. Similarly, the three and five women with PE in the DiPEP study who were respectively missed by the Geneva/D-dimer and YEARS/D-dimer strategies could have survived without adverse event if they had not been treated. A larger cohort of women receiving no treatment after negative assessment would be required to determine whether missed cases led to an unacceptable rate of adverse outcome.

It is an interesting observation that when using the van der Pol and modified Geneva scores, the five ‘missed’ PE were reported as small or segmental (see table 2). Moreover, these five had the lowest values of D-dimer. These two findings suggest that the volume of the emboli and/or lung tissue affected was small. Unfortunately, the techniques used to identify the PE cannot accurately assess the size of the thrombus and area of pulmonary damage. These observations tentatively suggest that the two scores do detect large PE. However, ignoring small PE is not a safe strategy as these may be a harbinger of later large PE.

The inconsistency between our study and previous studies is unlikely to be explained by differences in the study populations. The study populations had a similar prevalence of PE. The two previous studies were prospective and our analysis was limited to prospectively recruited pregnant women with suspected PE. The two previous studies both recruited women who would otherwise have received imaging (and the authors caution against extrapolating findings to other, lower risk, women) and our analysis was limited to those who received imaging. The van der Pol study described recruitment as consecutive, while the Righini and DiPEP studies did not. However, the recruitment rates across the studies suggested that the DiPEP population was not a more highly selected population, with 219 women recruited across eleven sites over 19 months, compared with 498 across 18 sites over 56 months recruited by van der Pol *et al* and 395 across eleven sites over 96 months recruited by Righini *et al.*

The DiPEP study had limitations that may explain the inconsistency with previous studies. Most women in the DiPEP study received anticoagulation prior to blood sampling. This reflects adherence to UK guidance^7^ that recommends immediate interim parenteral anticoagulant therapy if PE is suspected and imaging cannot be carried out immediately. The process of informing participants and acquiring consent in the DiPEP study meant that blood sampling could not be undertaken immediately. A pooled analysis by Couturaud *et al* 8 estimated that 24 hours after starting heparin therapy D-dimer levels have decreased by 25% in patients with acute VTE, while a more recent analysis by Baker and Keeling^9^ reported a mean decrease of 16% 12 hours after administration of low-molecular-weight heparin. This may have reduced the sensitivity of the strategies but the estimated reductions in D-dimer levels are insufficient to account for the false negative D-dimer results reported in table 4 and would not explain the false negative result in the patient who did not receive anticoagulation prior to blood sampling.

Another limitation of the DiPEP study is that the strategies were applied in theory but not in practice, and the assessment of PE probability was determined according to the documented diagnostic impression. This may be a source of bias if clinicians did not look for or record features such as unilateral lower limb pain, or if interpretation of the documented diagnostic impression is inaccurate. Furthermore, if clinicians are risk-averse, they may deviate from diagnostic protocols in a way that enhances sensitivity at the expense of specificity. For example, many clinicians would not be reassured by a negative D-dimer in the presence of a history of haemoptysis (patient 10) and would over-ride the diagnostic strategy and arrange imaging for PE in this case. The van der Pol and Righini studies have the advantage of showing what clinicians actually do in practice but may be undermined if clinicians exclude eligible patients from the study when they decide to over-ride the strategy.

The recruitment rates in these three studies raise concerns about whether the benefits of using clinical probability assessment and D-dimer to rule out PE are worth the risks. The recruitment rates suggest that less than five women per site per year would avoid imaging. The small cost savings and reduction in radiation risk associated with avoiding a small number of scans per year do not seem to justify the potentially catastrophic consequences of missing PE. For the fetus, the increased risk of childhood cancer to the age of 15 is 0.0006%, while for a mother with a typical background risk of developing breast cancer of 0.1% in the following 10 years, the absolute risk increase from 10 mGy of radiation is 0.0136%.^10–14^ Decision analytical modelling undertaken as part of the DiPEP study^15^ suggested that a clinical decision rule to avoid scanning in pregnant and postpartum women with suspected PE would need sensitivity >97.5% and specificity >90% to be cost-effective compared with a strategy of scanning for all.

## Conclusion

We have shown that the combination of clinical probability assessment and D-dimer does not reliably rule out suspected PE in pregnancy. It is uncertain whether PE missed by these strategies lead to clinically important consequences. Recent guidelines from the European Society for Cardiology and European Respiratory Society^15^ suggest using clinical probability and D-dimer to rule out PE in pregnancy, based on the Righini and van der Pol studies. Our findings should be taken into account when considering these guidelines.
